# Endothelin-3 is epigenetically silenced in endometrioid endometrial cancer

**DOI:** 10.1007/s00432-022-04525-w

**Published:** 2022-12-21

**Authors:** Nikola Zmarzły, Szymon Januszyk, Paweł Mieszczański, Emilia Morawiec, Paulina Buda, Konrad Dziobek, Marcin Opławski, Dariusz Boroń

**Affiliations:** 1 Department of Histology, Cytophysiology and Embryology, Faculty of Medicine in Zabrze, Academy of Silesia in Katowice, Zabrze, Poland; 2 ICZ Healthcare Hospital in Zywiec, Zywiec, Poland; 3 Hospital of Ministry of Interior and Administration, Katowice, Poland; 4 Gyncentrum Fertility Clinic, Katowice, Poland; 5 Department of Microbiology, Faculty of Medicine, University of Technology, Academy of Silesia in Katowice, Zabrze, Poland; 6 Department of Gynecology and Obstetrics With Gynecologic Oncology, Ludwik Rydygier Memorial Specialized Hospital, Krakow, Poland; 7 Department of Gynecology and Obstetrics, Faculty of Medicine and Health Sciences, Andrzej Frycz Modrzewski University in Cracow, Cracow, Poland; 8 Department of Gynecology and Obstetrics, TOMMED Specjalisci Od Zdrowia, Katowice, Poland; 9 Department of Gynecology and Obstetrics, Faculty of Medicine, University of Technology, Academy of Silesia in Katowice, Zabrze, Poland

**Keywords:** Endometrial cancer, Endothelins, EDN3, miRNA, DNA methylation

## Abstract

**Purpose:**

Changes in the activity of endothelins and their receptors may promote neoplastic processes. They can be caused by epigenetic modifications and modulators, but little is known about endothelin-3 (EDN3), particularly in endometrial cancer. The aim of the study was to determine the expression profile of endothelin family and their interactions with miRNAs, and to assess the degree of *EDN3* methylation.

**Methods:**

The study enrolled 45 patients with endometrioid endometrial cancer and 30 patients without neoplastic changes. The expression profile of endothelins and their receptors was determined with mRNA microarrays and RT-qPCR. The miRNA prediction was based on the miRNA microarray experiment and the mirDB tool. The degree of *EDN3* methylation was assessed by MSP.

**Results:**

*EDN1* and *EDNRA* were overexpressed regardless of endometrial cancer grade, which may be due to the lack of regulatory effect of miR-130a-3p and miR-485-3p, respectively. In addition, *EDN3* and *EDNRB* were significantly downregulated.

**Conclusion:**

The endothelial axis is disturbed in endometrioid endometrial cancer. The observed silencing of *EDN3* activity may be mainly due to DNA methylation.

## Introduction

Endometrial cancer (EC) mainly affects peri- and postmenopausal women, but it is estimated that up to a quarter of cases occurs in premenopausal women (Rizzo et al. 2018). It is currently one of the most frequently diagnosed gynecological cancer in the world (Sung et al. 2021). Many classification systems are available, ranging from histological to genetic feature-based systems, but they are still not fully accurate (Raffone et al. 2021).

During carcinogenesis, neoplastic cells acquire new properties that enable the avoidance of immune system responses, self-sufficiency in terms of growth signals, or the ability to maintain angiogenesis, invasion of nearby tissues, and metastasis. Current research indicates the simultaneous presence of genetic and epigenetic mechanisms in cancer and their mutual influence, favoring cancer formation (De Carvalho et al. 2012). Epigenetic modifications, including DNA methylation and miRNAs acting as epigenetic modulator, are the subject of research in modern cancer diagnostics and therapy (Leaderer et al. 2011; Joyce et al. 2018).

Endothelin-1 (EDN1), endothelin-2 (EDN2), endothelin-3 (EDN3), and their type A (EDNRA) and type B (EDNRB) receptors are responsible for the modulation of apoptosis, cell proliferation and survival, as well as angiogenesis (Sticherling 2006; Grimshaw 2007; Barton and Yanagisawa 2008). Maintaining the balance between the activities of endothelin receptors determines gene expression, cell division and differentiation. In cancer cells, this balance is disturbed, resulting in tumor progression and invasion of nearby tissues (Wiesmann et al. 2009). The mechanisms participating in the regulation of endothelin activity involve DNA methylation and miRNAs (Welch et al. 2013; Wang et al. 2013; Xie et al. 2018; Mahdi et al. 2020).

Disruption of the endothelin axis affects the growth of many cancers, including endometrial, prostate, colon, breast, lung, kidney, ovarian, cervical, and brain cancer (Bagnato et al. 2005). Published experimental works focus on their effects on endothelin-1 (Tsai et al. 2015) and endothelin receptors (Chen et al. 2013; Uddin et al. 2019), but little is known about endothelin-3, particularly in endometrial cancer. The aim of the study was therefore to determine the expression profile of endothelins and endothelin receptors and their interactions with miRNAs, as well as to assess the degree of *EDN3* methylation in endometrioid endometrial cancer.

## Results

### Expression profile of endothelins and their receptors determined by mRNA microarrays and RT-qPCR

One-way ANOVA revealed that 9 out 10 mRNAs representing endothelins and their receptors on the microarray were differentially expressed in endometrial cancer compared to the control. Tukey's post hoc test showed that the number of mRNAs differentiating each cancer grade from the control was as follows: G1 vs C, 5 mRNAs, G2 vs C, 8 mRNAs, and G3 vs C, 8 mRNAs. EDN1 showed overexpression as endometrial cancer progressed, while changes in EDN2 levels were not statistically significant. In the case of EDN3, its expression decreased regardless of cancer grade. Similarly, EDNRA showed an increase in its levels throughout the course of the disease. In turn, EDNRB expression was decreased, but these changes were significant only in G2 and G3 endometrial cancer (Table 1).

**Table 1:** Expression profile of endothelins and their receptors determined with mRNA microarrays

mRNA	ID	Fold-change		
		G1 vs C	G2 vs C	G3 vs C
*EDN1*	218995_s_at	2.18*	2.44*	2.51*
*EDN2*	206758_at	1.19	1.23	1.28
*EDN3*	208399_s_at	− 1.67*	− 2.40*	-2.81*
	217154_s_at	− 1.33*	− 1.96*	− 2.36*
*EDNRA*	204463_s_at	3.46*	2.63*	2.64*
	204464_s_at	2.87*	2.55*	2.26*
	216235_s_at	1.91	2.64*	2.53*
*EDNRB*	204271_s_at	− 1.15	− 1.69*	− 1.53*
	204273_at	− 1.01	− 1.27	− 1.24
	206701_x_at	− 1.31	− 2.00*	− 1.76*

*ID* number of the probe, *C* control, *G* grade of endometrial cancer

*p < 0.05 vs C group

The obtained results were further validated with RT-qPCR. A Shapiro–Wilk test revealed that the data were not normally distributed. The Kruskal–Wallis and Dunn’s tests were then carried out. *P* value adjustment for multiple testing was performed using Benjamini–Hochberg false discovery rate correction. Table 2 presents the results of the statistical analysis along with the median, first (Q1), and third (Q3) quartiles.

**Table 2:** Values of descriptive statistics, Kruskal–Wallis and Dunn’s tests in endometrial cancer and control (*p* < 0.05)

Gene	Group	mRNA copies/μg total RNA			Kruskal–Wallis test	Dunn’s test
		Me	Q1	Q3		
*EDN1*	C	52,980.5	47,678.5	55,640.75	< 0.001	G1 vs C, *p* < 0.001 G2 vs C, *p* < 0.001 G3 vs C, *p* < 0.001
	G1	92,852	84,906	95,477		
	G2	95,436	89,675.5	96,417		
	G3	98,312	91,480	103,585		
*EDN2*	C	12,588	12,107.25	13,094	0.0476	NS
	G1	13,512	12,856.00	13,962.5		
	G2	13,125	12,631.50	14,065.5		
	G3	13,387	12,431.00	14,296		
*EDN3*	C	18,893	16,014.25	20,878	< 0.001	G1 vs C, *p* < 0.001 G2 vs C, *p* < 0.001 G3 vs C, *p* < 0.001 G3 vs G2, *p* = 0.026 G3 vs G1, *p* = 0.001
	G1	2187	1453	2769.5		
	G2	1410	1218	1816		
	G3	626	316	873		
*EDNRA*	C	39,050	37,596.25	41,456.25	< 0.001	G1 vs C, *p* < 0.001 G2 vs C, *p* < 0.001 G3 vs C, *p* < 0.001 G3 vs G1, *p* = 0.010 G2 vs G1, *p* = 0.004
	G1	109,290	106,354.5	112,900		
	G2	88,290	86,330	92,870		
	G3	91,210	88,965	93,720		
*EDNRB*	C	26,633.5	25,016.25	27,767.5	< 0.001	G2 vs C, *p* < 0.001 G3 vs C, *p* < 0.001 G3 vs G1, *p* < 0.001 G2 vs G1, *p* = 0.007
	G1	23,617	22,495	25,595		
	G2	18,500	16,987.5	20,209.5		
	G3	15,069	13,745	15,839.5		

*Me* median, *Q1* lower quartile, *Q3* upper quartile, *C* control, *G* grade of endometrial cancer, *NS* not significant

The results revealed the overexpression of *EDN1* and *EDNRA*, while levels of *EDN3* and *EDNRB* were reduced. Furthermore, changes in *EDN2* expression were statistically insignificant, which is consistent with the results of the microarray experiment.

### EDN3 methylation profile

**Fig. 1:**
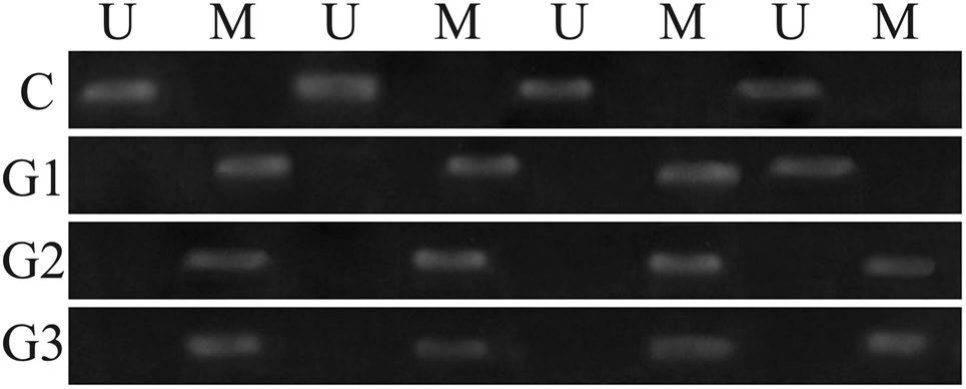
MSP results for selected samples. *U* use of primers for the unmethylated sequence, *M* use of primers for the methylated sequence

The electropherograms obtained after the separation of MSP products allowed for the assessment of the degree of *EDN3* methylation in the examined endometrial tissue samples. The presence of the band after the use of primers complementary to the methylated sequence indicates the presence of methylation, and in the case of primers for the unmethylated sequence, the appearance of the band indicates no methylation. Representative examples of the MSP are shown in Fig. 1

In the control group, methylation occurred in 1/30 (3.33%) cases. In G1 endometrial cancer, methylation was recorded in 11/15 (73.33%) samples. In G2 and G3 cancers, the methylation was observed in 13/15 (86.67%) and 14/15 (93.33%) cases, respectively (Table 3).

**Table 3:** The degree of methylation in the studied groups

Group	Methylated	Unmethylated
C	1 (3.33%)	29 (96.67%)
G1	11 (73.33%)	4 (26.67%)
G2	13 (86.67%)	2 (13.33%)
G3	14 (93.33%)	1 (6.67%)

*C* control, *G* grade of endometrial cancer

### miRNA target prediction

It was observed that 155 out of 1105 miRNAs found on the microarray demonstrated a significant change in expression. The number of miRNAs differentiating each cancer grade from the control was as follows: G1 vs C, 131 miRNAs; G2 vs C, 58 miRNAs; G3 vs C, 85 miRNAs. Then, it was assessed which of the differentiating miRNAs can participate in the regulation of *EDN1*, *EDN2*, *EDN3*, *EDNRA*, and *EDRNB* activity (Table 4).

**Table 4:** List of genes encoding endothelins and their receptors whose activity may be regulated by miRNAs in endometrial cancer

mRNA	Expression	miRNA	Fold-change		
			G1 vs C	G2 vs C	G3 vs C
EDN1	Increased	miR-130a-3p	− 32.26*	− 4.41*	− 13.63*
		miR-130b-3p	− 13.45*	1.23	1.51
		miR-4319	− 1.25*	− 1.15	− 1.03
		miR-379-5p	− 2.73*	− 2.26*	− 3.5
		miR-125b-5p	− 1.57	− 3.27*	− 6.55*
EDN3	Decreased	miR-520d-5p	1.04	1.09	1.34*
EDNRA	Increased	miR-30a-5p	− 6.84*	1.12	− 1.58
		miR-30c-5p	− 17.16*	1.64	1.62
		miR-30d-5p	− 3.51*	1.48	2.09
		miR-30e-5p	− 1.58*	− 1.41	− 1.41
		miR-539-5p	− 1.21*	− 1.25*	− 1.27
		miR-485-3p	− 1.38*	− 1.43*	-1.41*
		miR-197-3p	4.25*	1.45	2.93*
EDNRB	Decreased	miR-182-5p	− 4.6*	19.75*	122.79*
		miR-340-5p	− 1.17	− 1.19*	− 1.19
		miR-1271-5p	− 9.11*	− 3.06*	− 14.5*
		miR-567	1.24*	− 1.08	− 1.02

*C* control, *G* grade of endometrial cancer

*p < 0.05 vs C group

The obtained results indicate that *EDN1* overexpression may be the result of a significant decrease in miR-130a-3p activity, regardless of cancer grade. The low level of miR-130b-3p, miR-4319, and miR-379-5p in the early stage of endometrial cancer and the decreased expression of miR-125b-5p and miR-520d-5p in advanced cancer may also be important. Interestingly, there were no miRNAs that could potentially regulate *EDN2* expression. The low *EDN3* level may be the result of the gradually increasing expression of miR-520d-5p. In the case of *EDNRA*, its high expression may result from a reduction in the activity of miR-30a-5p, miR-30c-5p, miR-30d-5p, and miR-30e-5p in G1 cancer. Moreover, miR-485-3p showed low levels in all endometrial cancer grades. A significant drop in expression was also noted for miR-539-5p in G1 and G2 cancers, while miR-197-3p was overexpressed compared to the control. The decreased *EDNRB* level may be related to the low activity of miR-182-5p, miR-340-5p, miR-1271-5p, and miR-567.

## Discussion

DNA methylation is the most commonly described epigenetic modification affecting the level of gene expression. Under physiological conditions, it is responsible for the proper development of the organism, as it participates in the activation of the X chromosome in placental mammals, as well as the regulation of parental imprinting (Ghavifekr et al. 2013; Shevchenko et al. 2013). Normal cells have systems to protect their CpG islands against over-methylation; however, they may be avoided, resulting in hypermethylation and gene silencing, which in turn is important in tumor induction and progression (Long et al. 2016). It is worth mentioning that epigenetic modifications are mostly reversible, and therefore, they can serve as a potential target of drugs that restore the correct expression of individual genes (Tompkins et al. 2012).

With regard to methylation-based therapies, their strategy is based on the inhibition of DNA methyltransferases (DNMTs), which are responsible for establishing methylation patterns. Nucleoside analog inhibitors include 5′-azacytidine (Aza, Vidaza) and 5-aza-2′-deoxycytidine (Decitabine, Dacogen), which have been approved by the FDA for the treatment of myelodysplastic syndromes (Heuser et al. 2018). In addition, clinical trials are underway for Guadecitabine (SGI-110) in ovarian cancer, hepatocellular carcinoma, acute myelogenous leukemia, and myelodysplastic syndromes (Bennett and Licht 2018). Nonnucleoside analog inhibitors include hydralazine, mitoxantrone, procaine, and procainamide. They can reactivate some tumor suppressor genes and block the action of DNA methyltransferases (Hu et al. 2021). Antisense oligonucleotides, which are artificial nucleic acid polymers, are also used in the therapy. MG98, which blocks the activity of DNMT1, has the potential to inhibit tumor growth and reactivate tumor suppressor genes (Plummer et al. 2009; Amato et al. 2012).

In our study, the expression profile of endothelins and their receptors was assessed using mRNA microarrays, and then successfully validated by RT-qPCR. Particular attention was paid to *EDN3*, for which the methylation profile was additionally determined. Potential interactions of endothelins and their receptors with miRNAs were also evaluated.

Endothelins are characterized by a strong vasoconstrictor effect, but also a positive effect on cell growth and differentiation. They are also of importance in cancer development (Weydert et al. 2009). Endothelin-1 is a well-known growth factor secreted by tumor cells, regulating cellular processes through signaling via mitogen-activated protein kinase (MAPK), protein kinase B (Akt), integrin-linked kinase (ILK), and proto-oncogene tyrosine-protein kinase Src pathways (Teoh et al. 2014). Zhang et al. (2017) confirmed that silencing endothelin-1 can inhibit proliferation and invasion of lung cancer cells. Boldrini et al. (2005) showed that high EDN1 expression correlates with a poor prognosis in non-small cell lung carcinoma. Similar observations were made by Mitrakas et al. (2015) for non-metastatic muscle-invasive bladder cancer. In turn, Weydert et al. (2009) demonstrated increased secretion of endothelin-1 in prostate cancer cell lines (PC-3 and 22Rv1), and emphasized the importance of its local concentration and its impact on tumor growth, especially metastatic. EDN1 overexpression has also been reported in colon cancer (Kim et al. 2005), pancreatic cancer (Gupta et al. 2020), and ovarian cancer (Rosanò et al. 2014).

In our study, EDN1 showed a significant increase in expression regardless of the endometrial cancer grade in both the microarray and RT-qPCR experiments. MiRNA prediction has identified several molecules that may interact with EDN1. Interestingly, these miRNAs were characterized by reduced activity, which may suggest that their regulatory effect on EDN1 is absent, which results in its overproduction. We observed that miR-130a-3p expression was significantly decreased in all grades of endometrial cancer, with the greatest reduction in the initial stage of the disease. Roman-Canal et al. also reported a reduction in the level of this miRNA in the study of extracellular vesicles from the peritoneal lavage of EC patients (Roman-Canal et al. 2019). MiR-130a-3p is believed to have anti-cancer properties as described in colorectal cancer (Song et al. 2021), breast cancer (Kong et al. 2018; Poodineh et al. 2020), gastric cancer (Jiang et al. 2015), hepatocellular carcinoma (Li et al. 2014), glioblastoma (Wang et al. 2019), and nasopharyngeal carcinoma (Chen et al. 2017). Fan et al. (2021) also found that miR‑130a‑3p may promote proliferation and invasions in cervical cancer. In our study, a low activity was also observed for miR-130b-3p, miR-4319, and miR-379-5p in the early stage of endometrial cancer, which may be associated with the highest EDN1 expression in G1 cancer. Interestingly, miR-130b-3p can promote the progression of colorectal cancer (Song et al. 2022), while miR-4319 inhibits it (Huang et al. 2019). A decreased level of miR-379-5p was reported in breast cancer (Yang et al. 2022). In addition, Liang et al. (2021) found that miR-379-5p can inhibit the epithelial–mesenchymal transition (EMT) in endometrial cancer as a result of its ability to suppress proliferation, invasion, and migration.

In the case of endothelin-2, we did not notice significant changes in its expression in endometrial cancer compared to control. On the other hand, Liu et al. (2021) used EDN2 along with other genes to develop a prognostic prediction model and recognized it as an oncogene in the EC. Bot et al. (2012) observed its high level in renal cell carcinoma. In turn, Grimshaw et al. (2004) noted the promotion of invasive abilities by EDN2 in breast cancer.

In our study, we focused particularly on endothelin-3 as information regarding its epigenetic silencing has emerged in other cancers, but not in endometrial cancer. Wang et al. (2013) investigated the methylation level in colon cancer via methylation-specific PCR and concluded that EDN3 could be a potential target of epigenetic therapy. This was also confirmed by Olender et al. (2016) in colorectal cancer. Wiesmann et al. (2009) also reported downregulation of EDN3 as a result of methylation of its promoter in breast cancer. EDN3 level was also decreased in cervical cancer (Lin et al. 2017; Yang et al. 2020). Gargya et al. (2021) found that high EDN3 levels are associated with a low-risk endometrial cancer phenotype. The microarray experiment and RT-qPCR in this study indicated that EDN3 is downregulated in all endometrial cancer grades. MSP carried out in this study revealed that this may be due to DNA methylation. In addition, we also made a miRNA prediction to see whether the observed silencing of EDN3 expression could be the result of these two mechanisms. The change in activity was noted only for miR-520d-5p, whose expression gradually increased, but it was significant only in G3 cancer. Interestingly, miR-520d-5p is considered a suppressor of many cancers, including colorectal cancer (Yan et al. 2015), breast cancer, nasopharyngeal carcinoma (Tsukerman et al. 2014), and cervical cancer (Zhang et al. 2020). The increase in its expression in our study may suggest that the decrease in EDN3 level is mainly related to DNA methylation, and not miRNA activity. It is possible, however, that not all miRNAs that interact with EDN3 were present on the microarray.

The type A endothelin receptor preferentially binds with endothelin-1 and -2, while the type B receptor binds to the three endothelins with equal affinity (Halaka et al. 2020). In this study, EDRNA was overexpressed, especially in G1 cancer same as EDN1, while EDNRB levels were decreased in endometrial cancer compared to the control. Endothelin signaling is involved in a number of processes related to tumor growth and its progression, including cell survival, invasion, and metastasis (Bagnato et al. 2008). Papanikolaou et al. (2017) demonstrated EDN1 and EDNRA overexpression in prostate cancer which correlated with SNAIL activity, indicating possible EMT induction via the endothelin pathway. Similar conclusions were also drawn in the case of endometrial cancer (Czerwiński et al. 2021), where EDN1 and EDNRA levels were elevated, which is consistent with our observations. In the case of EDNRB, Liu et al. (2020) showed that its high level favors disease-free survival time in triple-negative breast cancer. In turn, its low expression may be associated with extracellular signal-regulated kinase 1 (ERK) signaling and lead to worse survival of patients with lung adenocarcinoma (Wei et al. 2020). On the other hand, Vasaikar et al. (2018) observed EDNRB overexpression in glioblastoma. The miRNA prediction indicated that miR-485-3p, whose activity was reduced in all endometrial cancer grades, may be involved in the regulation of EDNRA expression. Low levels of this miRNA favored the promotion of proliferation and migration in glioblastoma cells (Zhang et al. 2019). On the other hand, its overexpression inhibits metastasis of breast cancer (Lou et al. 2016) and the development of colorectal cancer (Taherdangkoo et al. 2020; Gurer et al. 2022).

This work allowed to determine the activity profile of endothelins and their receptors as well as to indicate potential miRNAs involved in the regulation of their expression. The degree of *EDN3* methylation was also assessed, which increased with the progression of endometrial cancer. Microarray analysis has been successfully validated by RT-qPCR. The obtained results of miRNA prediction revealed for the first time a potential relationship of differentiating miRNAs with endothelins and their receptors, but it is based on algorithms and not experimental data, which may be considered a weakness of the study.

The expression profile of endothelins and their receptors changes as endometrial cancer progresses, and epigenetic mechanisms and modulators may be involved. *EDN1* and *EDNRA* overexpression were observed throughout the course of the disease, which may be due to the lack of regulatory effect of miR-130a-3p and miR-485-3p, respectively. Low levels have been reported for *EDN3* and *EDNRB*. The mechanism responsible for the reduction of *EDN3* activity is mainly DNA methylation.

## Methods

### Patient samples

This study was approved by the Bioethical Committee operating at the Regional Medical Chamber in Krakow (185/KBL/OIL/2020 and 186/KBL/OIL/2020). All procedures involving human participants were performed in accordance with the guidelines of the 2013 Declaration of Helsinki. The confidentiality of the data and the anonymity of the patients were maintained at all times. Informed consent was obtained from all participants involved in this study.

The study group consisted of 45 patients diagnosed with endometrioid endometrial cancer (EEC) further divided into three equal subgroups according to the degree of histological differentiation (G1-G3). The control group included 30 patients without neoplastic changes who qualified for surgery due to the prolapse of the uterus. The exclusion criteria were as follows: endometriosis, non-endometrioid endometrial cancer, coexistence of another cancer, and the use of hormone therapy 24 months before surgery.

The obtained tissue samples were stored in tubes containing Allprotect Tissue Reagent (Qiagen GmbH, Hilden, Germany) according to the manufacturer's instructions. Total RNA was extracted with the TRIzol reagent (Invitrogen Life Technologies, Carlsbad, CA, USA). Genomic DNA extraction was performed using the QIAamp DNA Mini Kit (Qiagen GmbH, Hilden, Germany). The obtained DNA was bisulfite converted and purified with the EZ DNA Methylation-Gold Kit (Zymo Research, Irvine, CA, USA) according to the protocol provided by the manufacturer. The concentration and purity of the obtained extracts were assessed using a MaestroNano MN-913 nano-spectrophotometer (MaestroGen, Inc., USA).

### mRNA microarrays

Expression profile of endothelins and their receptors was determined using the GeneChip 3′IVT PLUS Reagent Kit (ThermoFisher Scientific, Waltham, MA, USA) and HG-U133A 2_0 oligonucleotide microarrays (Affymetrix, Santa Clara, CA, USA) according to the manufacturer’s instructions. Fluorescence signals were acquired by the GeneArray scanner (Agilent Technologies, Santa Clara, CA, USA). The methodology has been described in detail in other articles (Grabarek et al. 2021). Microarray experiment was further validated with RT-qPCR.

### RT-qPCR

SensiFast SYBR No-ROX One-Step Kit (Bioline, London, UK) was used to assess the expression profile of endothelins and their receptors. β-actin (ACTB) was an endogenous control. For every run, a standard curve was plotted based on which the mRNA copy numbers of studied genes were calculated. The curves were drawn based on β-actin at five different concentrations (400, 800, 2000, 4000, and 8000 copies of *ACTB* cDNA). Each run was completed by analyzing the melting curve of each sample to confirm the specificity of the reaction.

The thermal profile consisted of reverse transcription (45 °C, 10 min), polymerase activation (95 °C, 2 min), and 40 cycles of denaturation (95 °C, 5 s), annealing (60 °C, 10 s), and elongation (72 °C, 5 s). The results are presented as the mRNA copy number per 1 μg of total RNA. The sequences of the primers are listed in Table 5.

**Table 5:** Primers used in RT-qPCR

mRNA	RT-qPCR amplification primers (5′-3′)
*EDN1*	Forward: CCAATCTTGGAACAGTCTTTTCCT Reverse: GGACATCATTTGGGTCAACACTCC
*EDN2*	Forward: TTGGACATCATCTGGGTGAA Reverse: CTGTAGTGGCCCCTGTCTTG
*EDN3*	Forward: ATTGCCACCTGGACATCATT Reverse: GCAGGCCTTGTCATATCTCC
*EDNRA*	Forward: TTTGATGTGGCATTCAGCATACAGGTT Reverse: TGGCCTTTTGATCACAATGACTTT
*EDNRB*	Forward: CTGCATGCCACTTTTCTTTCTCAA Reverse: ACTGGCCATTTGGA-GGTGAGATGT
*ACTB*	Forward: TCACCCACACTGTGCCCATCTACGA Reverse: CAGCGGAACCGCTCATTGCCAATGG

*EDN1-3* endothelins 1–3, *EDNRA* endothelin receptor type A, *EDNRB* endothelin receptor type B, *ACTB* β-actine

### EDN3 methylation profile

The first step was to determine the location of the CpG islands in the *EDN3* sequence (NCBI Reference Sequence: NG_008050.1). The exon sequence starts at 5001 nucleotide, following the region containing the promoter. Using the MethPrimer program (http://www.urogene.org/cgi-bin/methprimer/methprimer.cgi; accessed on May 25, 2022), a CpG island (520 bp) was found on the border of this region and the first *EDN3* exon. Subsequently, primers allowing the detection of methylated and unmethylated sequences by PCR were designed with the following assumptions: CpG island length > 100 nucleotides, > 50% GC content, and ratio of observed to expected value > 0.6.

The DNA obtained after bisulfite conversion and purification was used in methylation-specific PCR (MSP) with the QuantiTect SYBR Green PCR Kit (Qiagen GmbH, Hilden, Germany) and the primers shown in Table 6. The amplification accuracy was confirmed by the positive control (methylated DNA) and the negative control (unmethylated DNA) of the EpiTect Control DNA kit (Qiagen GmbH, Hilden, Germany). The MSP was performed under the following thermal conditions: 95 °C pre-denaturation for 5 min, 40 three-step cycles (30 s each): 94 °C denaturation, 65 °C primer annealing, and 72 °C elongation.

**Table 6:** Characteristics of primers designed for the MSP

PCR amplification primers (5′-3′)		CG%	'C's
M	Forward: GGGTGGTGTAGAAGTTAGAAAAGTTC Reverse: AACTCCGACCAAAAATTAACTACG	61.56 70.83	5 8
U	Forward: TGGTGTAGAAGTTAGAAAAGTTTGA Reverse: AACTCCAACCAAAAATTAACTACAAC	56.00 73.08	5 9

*M* primers designed for methylated EDN3 sequences, *U* primers designed for unmethylated sequences, *'C’s* number of converted cytosines

After EDN3 amplification, the obtained products were separated by electrophoresis on a 1% agarose gel with the addition of ethidium bromide (final concentration 0.5 µg/ml) in 1×TBE buffer at 120 V. Analysis of the separated fragments was performed in the presence of pBR322/HaeIII as a size marker.

### miRNA analysis

The miRNA expression profile was assessed using the miRNA 2.0 microarrays (Affymetrix, Santa Clara, CA, USA). The FlashTag Biotin HSR RNA Labeling Kit (Affymetrix, Santa Clara, CA, USA) was used to label the RNA, the efficiency of which was verified by the ELOS QC assay. After hybridization, washing and staining with Hybridization Wash and Stain Kit (Affymetrix, Santa Clara, CA, USA), GeneChip Scanner 3000 7G (Affymetrix, Santa Clara, CA, USA), and Affymetrix GeneChip Command Console Software (AGCC) were used to acquire and analyze data.

The mirDB tool (http://mirdb.org; accessed on August 11, 2022) was then used to predict miRNAs targets among endothelins and their receptors (target score ≥ 70) (Chen et al. 2020).

### Statistical analysis

The results of the microarray experiments were analyzed with the Transcriptome Analysis Console software (Thermo Fisher Scientific, Waltham, MA, USA). One-way ANOVA and Tukey’s post hoc test were carried out (*p* < 0.05). Analysis of the RT-qPCR results was performed on R using RStudio (version 116 4.2.0, RStudio, Inc.). The normality of the data distribution was verified with the Shapiro–Wilk test. After confirming its absence, the Kruskal–Wallis and Dunn’s post hoc tests were carried out. *p* value adjustment for multiple testing was performed with the Benjamini–Hochberg false discovery rate correction.

## Declarations

### Data availability

Data are included in the manuscript.
